# Epistemic Injustice in Late-Stage Dementia: A Case for Non-Verbal Testimonial Injustice

**DOI:** 10.1080/02691728.2022.2103474

**Published:** 2022-09-20

**Authors:** Lucienne Spencer

**Affiliations:** Institute for Mental Health, School of Psychology, College of Life and Environmental Sciences, University of Birmingham, Birmingham, UK

**Keywords:** Epistemic Injustice, Testimonial Injustice, Dementia, Merleau-Ponty, Phenomenology

## Abstract

The literature on epistemic injustice has thus far confined the concept of testimonial injustice to speech expressions such as inquiring, discussing, deliberating, and, above all, telling. I propose that it is time to broaden the horizons of testimonial injustice to include a wider range of expressions. Controversially, the form of communication I have in mind is non-verbal expression. Non-verbal expression is a vital, though often overlooked, form of communication, particularly for people who have certain neurocognitive disorders. Dependency upon non-verbal expression is a common feature of some forms of neurocognitive disorders such as ‘intellectual disabilities’, autism and late-stage dementia. According to the narrow definition of testimonial injustice currently championed in the literature, people who express non-verbally are exempt from testimonial injustice. However, when we consider cases where meaningful communications from non-verbal people are dismissed or ignored in virtue of identity prejudice, there seems to be a distinct testimonial harm at play. Using late-stage dementia as a case study, I argue that the definition of testimonial injustice should be expanded to include all communicative practices, whether verbal or non-verbal, to encompass the epistemic harms inflicted upon some of the most marginalised in our society.

## Introduction

First-person reports play a critical role in diagnosing and treating psychiatric illness. In psychiatric healthcare, the healthcare professional relies on the patient’s testimony in order to diagnose and treat the illness at hand. Despite the profound significance of the patient’s voice in psychiatric healthcare, there is a growing concern for the ‘epistemic injustice’ occurring within psychiatric practice (Crichton, Carel, and Kidd 2017). In the case of psychiatric illness, epistemic injustice occurs when a speaker’s testimony is given lower credibility than it ought to (‘testimonial injustice’) or when their voice is excluded from the psychiatric interpretive framework (‘hermeneutical injustice’) due to identity prejudices attached to psychiatric illness (Crichton, Carel, and Kidd 2017). Emerging research on epistemic injustice in psychiatric illness has demonstrated that such unjust epistemic practices can lead to ignoring and even silencing those with psychiatric illness; this is doubly detrimental: not only to the medical task at hand but also to the person’s own sense-making of their psychiatric illness.

By exposing the epistemic harms that arise from sanist stigmatisation, the literature on epistemic injustice in psychiatry has already done much to amplify the voices of those with psychiatric illness. Yet, what about people who do not have a voice with which to speak in virtue of their illness? Persistent difficulties in the use of language (including spoken, written, or sign language) is a common feature of many neurocognitive disorders, such as what the DSM-5 refers to as ‘intellectual disability’, some forms of autism, and late-stage dementia (American Psychiatric Association 2013). If a neurodiverse person has no voice with which to give testimony, are they exempt from testimonial injustice? While it may appear counterintuitive, this essay will argue to the contrary. As a significant number of marginalised people cannot communicate verbally, I argue that we must urgently expand our definition of testimonial injustice to encompass all communicative practices, vocal or otherwise.

In section one, I begin by outlining the concept of testimonial injustice as it is defined in the literature today. In section two, drawing on Merleau-Ponty, I develop an account of non-verbal expression as a meaningful form of communication. By expanding Fricker’s concept of ‘testimonial sensibility’ to ‘communicative sensibility’, we can understand the ways in which non-verbal forms of communication (such as embodied gestures) show up in epistemic colour through this perceptual faculty. In section three, I use the case study of language inhibiting dementia to demonstrate how meaningful non-verbal expression may not register as epistemically charged if the viewer is influenced by identity prejudice. I dub this form of epistemic injustice ‘non-verbal testimonial injustice’. Finally, in section four, I use Fricker’s concept of ‘testimonial *justice’* to develop some corrective measures for non-verbal testimonial injustice. The path is then cleared for greater epistemic sensitivity toward those who rely upon non-verbal communication.

## What is Testimonial Injustice?

Epistemic injustice was first theorised by Fricker (2017, 53) to ‘delineate a distinctive class of wrongs, namely those in which someone is disingenuously downgraded and/or disadvantaged in respect of their status as an epistemic subject’. The *epistemic* nature of the injustice derives from a person being wronged in their capacity as a *knower*, as someone who can convey knowledge. To be clear, epistemic injustice is not present in all cases in which a person’s epistemic credibility is diminished. One can only be said to have suffered an epistemic injustice if the credibility deficit is rooted in unfair prejudice. For instance, I may doubt the reliability of a person’s testimony if they are a notorious liar. This would be a rational and fair reason to downgrade somebody’s credibility. What makes epistemic injustice distinct is that it is motivated by ‘identity prejudice’. Fricker (2007, 4) uses this term to refer to prejudice driven by socially instituted stereotypes – ‘a distorted image of the social type in question’. Fricker goes on to distinguish two forms of epistemic injustice: hermeneutical injustice and testimonial injustice. The latter is the focus of this essay.

Fricker (2012, 4) defines testimony as ‘human practices of telling, and accepting (or not) what we are told’; however, as Wanderer (2017, 32) observes, the term ‘testimony’ can be used to encompass several different speech-acts such as ‘inquiring, questioning, discussing, speculating and deliberating, as well as the myriad of thicker and culturally-specific forms that such activities take in differing contexts’. According to Fricker (2007, 86), we register a speaker’s testimony through a faculty of ‘testimonial sensibility’: a ‘perceptual faculty’ that allows the hearer to view a speaker in an ‘epistemically loaded’ way. Through the lens of this ‘social perception’, certain people appear epistemically reliable (Fricker 2007, 70). Identifying reliable and trustworthy informants serves our basic urge as epistemic agents to pool information and gain knowledge from our environment. However, persuasive prejudicial attitudes tend to throw this perceptual faculty off-kilter. Due to inherited prejudicial beliefs, people who belong to certain marginalised groups may fail to register as epistemically loaded within the hearer’s ‘testimonial sensibility’. In this instance, the marginalised speaker is vulnerable to testimonial injustice.

To draw out the salient features of testimonial injustice, Fricker introduces an example from *The Talented Mr Ripley*: Dickie has disappeared, and Marge Sherwood approaches Herbert Greenleaf (Dickie’s father) to express her suspicions that Tom Ripley murdered him. Greenleaf, who holds Ripley in high regard, responds by saying, ‘Marge, there’s female intuition, and then there are facts’ (Minghella 2000, 130). Greenleaf dismisses Sherwood’s claim that Ripley may have murdered Dickie as ‘female intuition’. This credibility deficit is grounded in an implicit prejudice against women’s ability to rationalise such matters. Fricker identifies this as an instance of *testimonial* injustice because the speaker’s testimony is given insufficient credibility based on the hearer’s prejudice.

Testimonial injustice is comprised of primary and secondary harms. The primary harm is the product of epistemic injustice more broadly, as it captures the harm of being undermined as an epistemic subject: ‘They are wrongfully excluded from participation in the practice that defines the very core of the very concept of knowledge’ (Fricker 2007, 145). As rational agents, undermining one’s capacity to give knowledge is to undermine something central to being human: ‘when someone suffers a testimonial injustice, they are degraded *qua* knower, and they are symbolically degraded *qua* human’ (Fricker 2007, 44). Explicitly, it is not merely the individual who suffers a deflated epistemic status but the marginalised social group that the individual seemingly represents.

In contrast, the secondary harm captures the practical ramifications that alter the life of those who experience systematic testimonial injustice. Fricker appeals to an example recounted by Linda Martin Alcoff, where an untenured philosophy professor ‘suffered two years of anguish and self-doubt’ when her epistemic credibility was deflated by her colleagues (Alcoff 2000, 248). The reduced epistemic status was caused by a white male graduate teaching assistant publicly questioning her competency. Fricker (2007, 49) observes that through a ‘prolonged erosion of epistemic confidence’, the speaker loses conviction in their epistemic capacity. She claims that, in time, repeated damage to one’s epistemic status can inhibit the development of the marginalised subject’s identity. Thus, testimonial injustice can lead to an erosion in 1) the subject’s human self-value and 2) the subject’s identity as a knower.

Since Fricker first introduced ‘testimonial injustice’ to the field, the term has been adopted enthusiastically and, consequently, has grown ever broader in scope. For instance, Wanderer distinguishes between ‘transactional testimonial injustice’ (whereby the act of telling someone something is obstructed due to a ‘breach’ in the social act of transaction) and ‘structural testimonial injustice’ (whereby entrenched social inequalities structurally bar certain marginalised groups from testimonial credibility) (Wanderer 2017).

Dotson adds to the concept of testimonial injustice that of ‘epistemic violence’: ‘a refusal, intentional or unintentional, of an audience to communicatively reciprocate a linguistic exchange owing to pernicious ignorance’. Drawing on Hornsby and Langton’s speech-act theory, Dotson attributes epistemic violence to a failure to take up the speaker’s speech-act in virtue of this pernicious ignorance. She identifies two forms of silencing that can arise after a prolonged experience of one’s speech act failing ‘uptake’ in this way. First, such epistemic violence can lead to ‘testimonial quieting’, whereby the marginalised speaker is no longer viewed as a knower. Alternatively, the marginalised speaker may truncate their own testimony ‘in order to ensure that the testimony contains only content for which one’s audience demonstrates testimonial competence’ (Dotson 2011, 238). Therefore, although the subject does speak, they edit their testimony to ensure that it is recognised as credible and receives its audience’s uptake. As such, the subject is coerced into a form of self-censorship.

Trip Glazer argues that Dotson’s concept of ‘epistemic violence’ can be taken a step further. Pernicious ignorance can lead not only to a violation of linguistic communicative exchange, but also non-linguistic, emotional exchange. When a non-linguistic expression of emotion is wilfully misinterpreted, the marginalised person can suffer what Glazer calls ‘emotional misperception’ (Glazer 2019). Glazer draws on various examples from feminist philosophy to illustrate ‘emotional misperception’; for instance, the tone of Black women is frequently misread as being angry due to racist stereotypes (West 2018). Glazer identifies this as a non-linguistic form of epistemic violence, as it is the behavioural signifier of tone (opposed to the speech expression itself) that has been wilfully misunderstood.^1^ In this case, an epistemic violence has occurred as pernicious ignorance has caused an emotion to be misinterpreted.

While ‘emotional misperception’ is a valuable contribution to the literature, I believe we can take a step further still. Through this paper, I identify instances where non-linguistic expressions are not simply misread but *not even registered as meaningful expressions at all*.^2^ I argue that certain marginalised groups, particularly those dependent upon non-verbal communication, fail to appear as ‘epistemically loaded’. As such, they are not seen as the kind of people who can make meaningful emotional expressions. Rather than incorrectly being interpreted as ‘angry’ or ‘upset’, their non-linguistic expressions are missed entirely by our faculty of ‘testimonial sensibility’. Just as a testimonial exchange can fail to register as meaningful if the speaker is not considered an epistemic agent, so too can a non-linguistic exchange fail to register as meaningful. Drawing on people with language-inhibiting dementia as a case study, I argue that the concept of testimonial injustice ought to be extended to include the downgrading of non-verbal expression.

## Non-Verbal Communication

Consider the following example: you are sitting on a busy commuter train to London. At the next stop, another person boards the train. He is soaking wet and bedraggled from the downpour outside. His face is scrunched up in a frown as he tries to remove his wet hair from his face with one hand while clinging to the luggage rack with the other. As the train starts to move again, he stumbles slightly, unsteady on his feet. His face shifts from an expression of frustration to one of concern as he notices the sea of heads filling almost every seat. At this point, you stand up; rather than shout across the noisy train, you simply gesture towards your seat and smile at the man. The man’s face shifts to an expression of relief. He nods politely and shuffles towards the now vacant seat.

In this commonplace scenario, a meaningful communication has taken place without one word being uttered. You do not need the man to say aloud, ‘I am frustrated’, ‘I am concerned’ or ‘I am looking for a seat’ in order to infer what his gestures mean. This is because non-verbal communication is an essential aspect of our interpersonal relationships. In fact, an estimated 60–65% of our communication is non-verbal (Burgoon, Guerrero, and Floyd 2010). It is perhaps for this reason non-verbal expression has received so much philosophical attention, particularly in order to overcome our solipsistic fears and answer the age-old question: how do we know that other beings are ‘thinking’ beings like myself? Much of this debate has been subsumed under the ‘theory of mind’ scholarship- a debate I do not intend to weigh in on here. My purpose for this paper is to convince the reader that we do not register people as ‘knowers’ through testimony alone. Non-verbal expression is another essential way we understand people to be ‘knowers’. I make this argument with recourse to Merleau-Ponty’s phenomenological method.

The object of phenomenology is the study of appearances. Naturally, appearances must appear *to* something, and phenomenologists identify this ‘something’ as subjectivity. Merleau-Ponty’s ([1954] 2012, 84) distinct phenomenological method locates subjectivity, not in the mind, but in the body, as the ‘vehicle of being in the world’. For Merleau-Ponty, the body is the infrastructure of all expressive activity: ‘expression … is Merleau-Ponty’s master term for a creative, productive cognitive power—a power that is rooted in the excess of embodied perceptual life’ (Hass 2008, 172). As such, the embodied subject outputs meaningful bodily expression to the world.

As embodied subjects, we operate within a world that we understand to be shared with other embodied subjects. We do not experience Others in the world in the same way as we do inanimate objects. We witness that Others in the world must possess a subjectivity like my own, in virtue of the fact that they engage with the world in much the same way as myself.^3^ Merleau-Ponty provides the following example:

I am watching this man who is motionless in sleep and suddenly he wakes. He opens his eyes. He makes a move towards his hat, which has fallen beside him, and picks it up to protect himself from the sun. What finally convinces me that my sun is the same as his, that he sees and feels it as I do, and that after all there are two of us perceiving the world [is as follows] … When the man asleep in the midst of my objects begins to make gestures towards them, to make use of them, I cannot doubt for a moment that the world to which he is orientated is truly the same world that I perceive. (Merleau-Ponty 1973 [1969], 136)

Merleau-Ponty recognises that he and the Other share a common world as they are simultaneously impacted by their environment, in this case, they are impacted by the blazing heat of the sun: the sun burns them, makes their eyes squint, makes them sweat and makes them raise their hand over their forehead in a protective gesture or reach for a hat. Merleau-Ponty (1973 [1969], 137) recognises in the Other’s gestures that they experience the same ‘bite of the world’. This symmetry is enough to convince him that the Other is moved and touched by the same world, and as such, is an embodied subject positioned in the world much like himself. We can conclude that the gestures of the Other are *meaningful* to us. Through his gestures, Merleau-Ponty understands that the man wants his hat, is hot, is tired, and so on. In line with Fricker, the man registers as ‘epistemically charged’ for Merleau-Ponty.^4^

Moreover, for Merleau-Ponty, when he sees the gesture of the Other he does not need to *infer* what the other embodied subject means to express. Using the example of a person shaking their fist in anger, Merleau-Ponty ([1945] 2012, 190) remarks: ‘the gesture does not make me think of anger, it is anger itself’. No inference is necessary; in the words of Gallagher and Zahavi (2012, 207), the gesture is ‘saturated with the meaning of the mind; it reveals the mind to us’, and in this sense, we experience the emotion as directly as we can without first-person access. Therefore, gesture does not merely signify meaning *but is meaning itself*. When we see the Other shaking their first in anger, we do not infer anger from their gesture but witness it directly. With recourse to Fricker, we can conclude that we pre-reflectively take up the Other’s gestural expression in a mode of ‘*critical openness’* whereby ‘this stance allows her to take in knowledge … effortlessly’ (2007, 66).

Therefore, much like the act of ‘telling’, gesture is a form of meaningful communication. In line with Fricker, I argue that in my example of the man on the train and Merleau-Ponty’s example of the man in the sun, both are perceived to be ‘epistemically loaded’ by the onlooker. In fact, if we did not register other people as ‘epistemically loaded’, we would never solicit information from them in the first place. In such scenarios, ‘testimonial sensibility’ seems too narrow. As a ‘perceptual faculty’ that allows us to view people in epistemic colour, I propose the term should be revised to ‘communicative sensibility’, with the understanding that information and knowledge can be communicated through both verbal and non-verbal expression.

In what situation can this ‘communicative sensibility’ be said to fail? One (potentially) amoral way in which this ‘communicative sensibility’ fails is through our encounters with non-human animals.^5^ Imagine witnessing two African elephants in a zoo, seemingly doing nothing of note. They simply make the typical behavioral movements one would expect an elephant to make. However, unbeknownst to you, their subtle movements are communicative gestures to one another. Through ear-waves, trunk dragging, tossing of the head or strutting, one elephant may signal to the other that they are ready to mate, that they want to play or that there has been a death in the herd. Yet, the gestures of the elephant are meaningless movements to the untrained eye. In this sense, the elephant does not register in our ‘communicative sensibility’ as ‘epistemically charged’. As a differently bodied animal, it encounters the world in a way that is alien to us; therefore, the entirely distinct meaning-structure of their species goes unrecognised by our ‘communicative sensibility’. In contrast, elephant experts have trained their ‘communicative sensibility’ to register such gestures as meaningful. For the elephant expert, these gestures ‘say’ ‘I am bored’, ‘I am ready to mate’, ‘I am sad’ and so on. However, through an untrained ‘communicative sensibility’, these gestures ‘say’ nothing.

In what follows, I explore a case in which a communicative act is missed by our untrained ‘communicative sensibility’ in a distinctly immoral way. In this case, the communicative act is not missed because the communicator is of an entirely different species. Although a fellow embodied human being, their communicative act does not register as ‘epistemically charged’ because their credibility has been downgraded. By expanding ‘testimonial sensibility’ to ‘communicative sensibility’, we can capture cases whereby a valuable non-verbal expression is dismissed as meaningless by virtue of an identity prejudice. I argue that the latter constitutes a case of what I call ‘non-verbal testimonial injustice’.

The non-verbal group that this paper focuses on are those who depend on non-verbal communication as a result of disability or neurodiversity. This includes people with autism, people with intellectual disabilities, stroke victims, and people with dementia. These groups are especially vulnerable to epistemic injustice due to the pathophobic attitudes latent in our society (Kidd 2019; Kidd and Carel 2017; Crichton et al. 2017). People who communicate non-verbally are often isolated and communicate predominantly with carers and family members. As such, the non-verbal communications of such groups are particularly fragile. It is therefore vital for those within their social circle to take their non-verbal communications seriously in order to have any kind of social interaction at all.

I have chosen to focus on non-verbal people with dementia because the context of their condition leaves them particularly susceptible to non-verbal testimonial injustice. First, people with dementia are subjected to both ageist and pathophobic prejudices. Urbańska et al. dub this intersectional prejudice of ageism and pathophobia the ‘double stigma of dementia’ (2015). Second, as people with dementia are often older, their gestures are commonly slower, shakier and altogether less confident than the gestures of younger people. I will argue that this gestural quality makes people with dementia even more vulnerable to credibility deficit. Although I focus on dementia as a case study here, I do not believe that people with dementia belong to a special epistemic category. The concept of non-verbal testimonial injustice can encompass all epistemic subjects who depend on non-verbal (non-linguistic^6^) communication.^7^

## Non-Verbal Testimonial Injustice and Dementia

### Testimonial Injustice in Dementia

The umbrella term ‘dementia’ captures a form of major neurocognitive disorder that leads to a decline in neuro-normative cognitive functioning. This includes difficulties with information processing, language skills, and memory. Some common forms of dementia include Alzheimer’s disease, vascular dementia, dementia with Lewy bodies, frontotemporal dementia, and mixed dementia. According to the World Alzheimer’s Report 2018, around 50,000,000 people live with dementia worldwide. This number is projected to rise to 152,000,000 by 2050 (Patterson 2018). Despite these numbers, people with dementia often suffer credibility-deflating identity prejudice attributed to both ageist attitudes (the risk of dementia rises steeply with age) and the stigma attached to the illness itself (Evans 2018).^8^ People with dementia are often perceived to be ‘stupid’, ‘child-like’, ‘crazy’, ‘not all there’, and ‘not themselves anymore’. Such identity prejudice suggests that people with dementia are ‘incapable of purposeful and meaningful communication and the pursuit of life-enhancing relationships and activities’ (Kontos et al. 2020). The work of epistemic injustice scholars such as Jongsma et al. (2017) and Young et al. (2019) demonstrate how such pathophobic attitudes can fuel a credibility deficit towards people with dementia so that their testimony is considered unreliable, confused, or altogether meaningless.

For instance, Jongsma, Spaeth, and Schicktanz (2017) conducted a series of interviews to uncover the credibility deficit suffered by people with dementia in patient organisations. The interviews suggested that solicited testimony from dementia patients is often merely ‘tokenistic’; while they may appear to be heard in a formal context, their testimony bears little or no weight on decision-making processes:

This is an indication that affected members are cast as passive bystanders not only with regard to the decision-making process within [patient organisations], as we have seen before, but also with regard to their testimonies in respect to political representation. (Jongsma, Spaeth, and Schicktanz 2017, 228)

Crichton et al. (2017) and Young et al. (2019) posit that such deflated credibility may be attributed to an ‘anticipation of future loss’; in other words, the testimony of a person with dementia may be downgraded because it is assumed that the person has progressed to a later stage of the dementia process than they actually have (Young et al. 2019, 79). This credibility judgement is unfounded because such severe cognitive dysfunction ‘is hardly ever the case, except perhaps in the final stage of the illness’ (Crichton, Carel, and Kidd 2017).

These authors have done much to illuminate the different ways in which testimonial injustice can emerge from the distinct, negative identity prejudices that track people with dementia. Yet, while they prudently address the testimonial injustice that arises from the prejudicial assumption that people with dementia cannot speak for themselves (even though they have not yet reached that stage of the disease process), they fail to address whether epistemic injustice arises in cases where the person with dementia really has lost the capacity of speech. The reason for this omission may be that people with language-inhibiting dementia are seemingly automatically excluded from epistemic practices as their illness prevents them from participation, regardless of whether they are subjected to an identity prejudice or not. In what follows, I correct this assumption, arguing that to assume that non-verbal people with dementia are beyond the remit of testimonial injustice is to commit the very credibility deflation that the literature has sought to overcome. I argue that a kind of testimonial injustice is inflicted upon people with language-inhibiting dementia if their non-verbal communication is ignored or disregarded as meaningless. I call this *non-verbal testimonial injustice*.

### Non-Verbal Testimonial Injustice in Dementia

Loss of language is a common feature of dementia: ‘Patients with dementia demonstrate, among other signs, word-finding problems (anomia), sentence comprehension deficits, and lack of cohesion in discourse’ (Kempler and Goral 2008, 73). Depending on the stage of the dementia process, language dysfunction can vary. A dementia patient may retain verbal expression but experience language impairment, such as problems with phonology (e.g. saying ‘tair’ instead of ‘chair’), syntax (e.g. ‘the cookies must be pretty good they’re eating’) or vocabulary (e.g. saying ‘bench’ instead of ‘stool’) (Cummings 2020). At later stages of dementia, patients may lose the capacity for verbal expression altogether.

Some may argue that, due to such cognitive decline, people with dementia naturally lose their status as ‘knowers’ and therefore cannot be subject to a distinctly *epistemic* kind of injustice. Such pre-emptive objections call us to review Fricker’s definition of an epistemic subject; for Fricker (2007, 100), an epistemic subject is someone who has the capacity ‘to participate in an epistemic practice whereby information is shared or pooled’. Do people with language-inhibiting dementia possess this capacity? We can answer this in the affirmative. Studies show that non-verbal dementia residents can communicate their needs, and preferences and often have the capacity to participate in the decision-making process if taken seriously (Cameron et al. 2020; Ellis and Astell 2017; Vries 2013; Kontos 2011). People with language-inhibiting dementia can communicate that they are in pain (by pointing to the area of their body causing pain, or wincing), if they want or don’t want something (pointing, pulling towards, pushing away), dis/agreement (nodding, shaking head, shrugging shoulders, a dismissive flick of the wrist), emotion (laughing, sighing, tutting, eyebrow raising, smiling, frowning, winking, blowing kisses, clapping hands, hand squeezing) and express themselves in artistic ways (drawing, painting, music, singing, dancing) (Cameron et al. 2020; Ellis and Astell 2017; Vries 2013; Kontos 2011). Therefore, people with language-inhibiting dementia still retain important epistemic capacities that allow them to participate as epistemic agents.

For people with language-inhibiting dementia, non-verbal communication is essential. Non-verbal expression may take the form of the aforementioned art therapies such as drawing, painting, drama, music, and poetry. However, the most common form of communication for non-verbal dementia patients is touch, body language, hand gesture, and facial expression. For an illustration of meaningful non-verbal expression, consider the following example from a film clip presented by Kontos (2010) of a ‘virtually non-verbal’ person with dementia, Gladys, communicating with a healthcare practitioner, Naomi. The following is a segment of Zeiler’s description of the interaction:

Naomi has entered the room. She faces Gladys, leans forward towards her, touches her hand and greets her with the words “‘Mrs Wilson. Hello’.” Gladys doesn’t open her eyes, but she reaches forward to Naomi’s arm and holds it while Naomi sits down. Naomi asks “‘Can you see me good?’” At this, Gladys opens her eyes a bit, closes them again, holds on to Naomi’s hand and moves her own and Naomi’s hand up and down, repetitively … Moving very close to Gladys, Naomi says that she can see a tear in Gladys’s eyes. She touches Gladys’s cheek gently, first with one hand and then with both hands, and asks “‘Can you let me in a little bit?’” She then passes her hand over Naomi’s hand, arm and cheek. At this, Gladys straightens herself up and starts to clap her hand fast to the armchair, regularly and audibly. Naomi says “‘I think I can be with you and Jesus for a minute’” and she starts to sing “‘Jesus loves me, yes I know … ’” At this, Gladys slows down the pace of her hand clapping the armchair, and attunes the beat to the pace of Naomi’s songs. (Zeiler 2014 135–136)

Zeiler draws upon the example of Gladys and Naomi in order to demonstrate the power of joint musical activity for interaction with non-verbal people. However, the embodied interaction leading up to the musical engagement, and the musical engagement itself, can also be used to broadly demonstrate meaningful non-verbal communication from a person with dementia. Applying the account of ‘communicative sensibility’ established in the previous section, we could claim that Naomi’s ‘communicative sensibility’ evidently registers Gladys as ‘epistemically charged’. Consequently, Gladys’s gestures are coloured in such a way that they appear meaningful to Naomi. In a Merleau-Pontian sense, Naomi recognises Gladys as an embodied subject, making gestures that *are* the emotions she means to convey. Through Gladys’ clapping hands, for instance, Naomi can directly perceive her joy. Due to Naomi’s unprejudiced ‘communicative sensibility’, Gladys has had a meaningful interaction with Naomi through non-verbal expression.

But what if Naomi’s ‘communicative sensibility’ were not so well trained? Imagine if Naomi had been influenced by inbuilt societal prejudices towards those with language inhibiting dementia that portray people like Gladys as ‘child-like’, ‘stupid’ or ‘in a vegetative state’. Instead of recognising Gladys’ non-verbal expressions as a form of communication, Naomi’s ‘communicative sensibility’ would disregard Gladys’ gestures as meaningless behavioural patterns from someone who lacks the cognitive capacity for communication. By virtue of the prejudice attached to Gladys’ identity, she may not appear ‘epistemically charged’. If this were the case, Naomi might have instead perceived Gladys’ clasping of her arm and banging on the chair as inane bodily reflexes that communicate nothing. Consequently, her gestures would likely be ignored, and questions would be directed toward a family member or carer instead of Gladys herself.

Unfortunately, such one-sided communication from non-verbal people with dementia is not uncommon. In the context of care homes, studies show that many social interactions between staff and dementia residents are rushed and infrequent (Fauth, Meyer, and Rose 2020; Machiels et al. 2017; CQC 2014; Ballard et al. 2001). A study conducted by Fauth, Meyer, and Rose (2020, 763) found that dementia residents in care homes were given ‘no interaction’ 80.7% of the time. Recent Care Quality Commission (CQC) inspection reports support these findings and show that social interaction is even less common for residents with non-verbal communication. In fact, multiple reports noted that care homes that claim to specialise in dementia care had no methods in place for communicating with their non-verbal residents (2022a, 2022b, 2021a, 2021b, 2021c). According to one report:

We observed people who could fully communicate were provided with choices by staff. However, [non-verbal residents] were not provided with choices … The registered manager confirmed some people required pictorial aids to communicate and understand information, but these had not been sourced for staff to use. (CQC 2021c)

This led to instances where the needs of residents were not being met:

We [the inspectors] used ‘talking mats’ to communicate with one [non-verbal] person. Talking mats is a way for people who cannot verbally communicate to provide their feedback via pictorial aids. The person told us they did not like their bedroom or their food. Staff were unaware of this. Staff highlighted the person moved their bed every night, which staff moved back every morning and had not considered the person might prefer their room that way. (CQC 2021c)

CQC reports show that non-verbal residents were more likely to be interacted with in a merely task-focused manner (i.e. feeding, getting out of bed, medicating etc.) without communication:

We [the inspectors] observed how a staff member wiped a person’s face after eating. They did not ask the person or offer them the opportunity to wipe their own mouth but instead bent forward and wiped their face. The person was visibly startled by this and pulled away showing they did not enjoy the experience. A similar reaction was observed with a second person, but the staff member did not appear to notice people’s discomfort. (CQC 2022b)

We saw two staff go into one room, put the light on and start getting [a non-verbal resident] up by removing their duvet, without allowing the person to wake up, asking for their consent or how they were. They said, ‘We’ve come to get you up’. (CQC 2021a)

The de-prioritisation of non-verbal residents could also be observed through the structure of the care home itself:

Some rooms had little décor or pictures and no information to assist in getting to know the person. Other rooms, however, were warm and cosy and personalised. We found that the rooms with little or no personalised effects belonged to people in the later stages of dementia. This suggested that people with higher needs, or in the later stages of dementia, did not have their dignity maintained in the same way as people who were able to make choices and advocate their own needs. (CQC 2014)

One person who stayed in their room was described by a member of staff as having ‘closed down’. We found their room was the furthest away from the lounge and the person was not provided with any method of summoning help. (CQC 2014)

These CQC-conducted inspections indicate that non-verbal people with dementia are frequently given less attention than they deserve and are less likely to be engaged in meaningful social interaction. Non-verbal people with dementia are likely to be missed by such ‘communicative sensibility’ because it is ‘more challenging to see and recognize individuals with dementia as relational human beings when they cannot respond verbally’ (Heggestad, Nortvedt, and Slettebø 2015, 835). I suggest that in this instance, we ought to understand this harm as a form of non-verbal testimonial injustice.

According to Fricker (2007, 20), ‘the idea is to explore testimonial injustice as a distinctively *epistemic* injustice, as a kind of injustice in which someone is *wronged specifically in her capacity as a knower*’. I argue that in the case of non-verbal testimonial injustice, a marginalised person has indeed been undermined in their status as someone who can participate in knowledge exchanging practices. Following Merleau-Ponty’s account of meaningful gesture, knowledge and information are pre-reflectively conveyed through non-verbal communication. Consequently, to assume that people who can only communicate non-verbally cannot be ‘knowers’ is surely epistemically unjust. If a non-verbal marginalised knower does not register as ‘epistemically charged’, their communicative gestures are likely to be met with a credibility deficit. For people with language inhibiting dementia, this credibility deflation can be systematic and persistent, as the stigma launched against them tracks them through several different social domains. As such, their non-verbal communications may be repeatedly dismissed by carers, GPs, the police, lawyers, family members, and so on.

In the words of Fricker (2007, 160), ‘the characteristic expressive style of a given social group may be rendered just as much of an unfair hindrance to their communicative efforts’. In her discussion on testimonial injustice, Fricker suggests that the accent of the speaker may impact the credibility ascribed to a given testimony:

Not only does accent carry a social charge that affects how a hearer perceives a speaker (it may indicate a certain educational/class/regional background), but very often it also carries an epistemic charge. Accent can have a significant impact on how much credibility a hearer affords a speaker, especially in a one-off exchange. (Fricker 2007, 17)

If the credibility deficit attached to a person’s testimony may be influenced by their vocal style, we can reasonably conclude that, in the case of non-verbal expression, a credibility deficit may be influenced by *gestural style*.

Compare the following two examples of the same gesture. The first example is that of a nurse outside a hospital. Holding your gaze, he points firmly to the cigarette in your hand and then shakes his head. You understand this to mean that you are smoking in a non-smoking area, so you put out your cigarette. In the second scenario, the gesture is carried out by a person with language-inhibiting dementia. They, too, point to the cigarette in your hand and shake their head. However, their movement is slow, shaky, and less precise. Their movements are so slow that you might not give them time to finish their gesture before you turn away, dismissing their gesture as meaningless. If you *do* take the time to watch the person’s gesture, you may assume that their unsteady pointing and uneasy head shaking are either meaningless movements or merely a product of a delusion (perhaps you think that you have been mistaken for someone else: judging from the shaking of their head, someone they disliked). Either way, the gesture is unduly ignored or dismissed. Like an accent, a gesture can carry an epistemic charge. Whether slow and uncertain or firm and precise, the gestural style is likely to influence the speaker’s credibility. For instance, studies show that handshakes with ‘strength, vigor, duration, eye contact, and completeness of grip’ are more likely to convey confidence, trustworthiness, and experience to the recipient (Chaplin et al. 2000). Therefore, it is not unreasonable to suggest that slow, shaky, and imprecise gestures would convey untrustworthiness and unreliability. Therefore, I argue that not only the gesture itself, but the gestural style that may deflate the marginalised knower’s epistemic status.

Finally, I propose that those subjected to non-verbal testimonial injustice are also vulnerable to a kind of pre-emptive testimonial injustice. Fricker observes that once a group of people are labeled as ‘unreliable’, there is a risk that they will be bypassed altogether before an interaction has even taken place. Rather than addressing an unreliable person and dismissing their testimony as untrustworthy, the marginalised group of unreliable people are simply not approached for information in the first place (2007, 130). Fricker refers to this bypassing as ‘pre-emptive testimonial injustice’ as ‘the speaker is silenced by the identity prejudice that undermines her credibility in advance’ (130).

Although not her intention, Sally Haslanger provides a useful example of silencing those marginalised in our society in advance of a speech expression. She recounts the following experience:

Recently in an airport next to my husband who is in a wheelchair, an airline employee asked me: ‘What is your husband’s name’? as if the fully alert individual in the wheelchair in front of him couldn’t answer even that simple question on his own. (Haslanger 2017, 285–286)

Here, the airline employee has been influenced by prejudice against the epistemic capacity of disabled people. Subsequently, the airline employee bypassed Haslanger’s husband altogether and directed his question towards Haslanger herself, who is afforded epistemic privilege as a non-disabled person. Haslanger’s example exposes an instance of pre-emptive testimonial injustice as her husband is excluded from the conversation in advance due to a credibility deficit.

In what way can someone who can only communicate non-verbally (and therefore cannot ‘answer even that simple question on her own’) be pre-emptively *silenced*? I argue that a non-verbal person may be pre-emptively silenced if they are excluded from social interaction in advance. Recent studies show that non-verbal people with dementia in care homes are often excluded from residential activities because they are perceived to be unable to take part: ‘when a person could not participate in the social activities provided, other options were seldom offered’ (Melander et al. 2018, 6). If a person is excluded from social activities and, therefore, social interactions in advance, they are pre-emptively silenced. A more straightforward way pre-emptive silencing can occur is if a non-verbal person is not even observed. A study conducted by Heggestad et al. (2015) recorded several instances where non-verbal residents were ignored by staff in a way that meant non-verbal communication could not even get off the ground. For instance, one relative observed the following:

I have seen some examples where … they are sitting and feeding a person, and while they are feeding him or her, they have been talking to someone else. And I have seen situations where one of the carers just mechanically put something into the resident’s mouth. It’s terrible! I’ve also seen other examples where they talk to a relative over the resident’s head. (Heggestad, Nortvedt, and Slettebø 2015, 835)

Consider, again, Zeiler’s example of Gladys (the person with language inhibiting dementia) and Naomi (the practitioner). Imagine if, instead of engaging with Gladys, Naomi never even looked at her. Imagine Naomi walked into the room and spoke with the other patients who can communicate verbally yet did not even glance at Gladys because she knows Gladys cannot communicate verbally. If Naomi does not look at Gladys due to the prejudicial assumption that Gladys cannot communicate anything meaningful in the first place, then I propose that Gladys’s nonverbal communication has been pre-emptively silenced.

Non-verbal communication cannot occur unless someone looks in the marginalised person’s direction and witnesses their gestures. Without this basic interaction, a non-verbal expression is silenced before the perceptual faculty of ‘communicative sensibility’ can kick in. Given the pathophobic prejudices attached to language inhibiting dementia, one in three people admit to avoiding people with dementia because their condition makes them feel uncomfortable (Department of Health 2010). This suggests that those with advanced dementia are frequently not even looked at and are therefore vulnerable to pre-emptive silencing.

### The Harm of Non-Verbal Testimonial Injustice

What harm can result from non-verbal testimonial injustice? There is a primary harm in the first instance, whereby a person is robbed of an essential human value: their status as a giver of knowledge (Fricker 2007, 44). This intrinsic harm renders the marginalised person as something less than human. This harm is all the more apparent when we consider Merleau-Ponty’s account of gesture. Recall, for Merleau-Ponty, when we witness another person’s gesture as meaningful, we understand them to be an embodied being just like us. In contrast, those whose gestures we regard as uncommunicative and devoid of sense are outside the realm of embodied, communicative beings. Merleau-Ponty criticizes the manner in which certain groups, ‘animals, children, madmen and primitive peoples,^9^ are often wrongly excluded from our scope of intersubjectivity:

either the being that stands before us may be likened to a human being, in which case it can be given, by analogy, the usual human attributes of the healthy adult. Alternatively, it is no more than a blind mechanism—living chaos—in which case meaning cannot possibly be ascribed to its behaviour. (Merleau-Ponty [1948] 2008, 44)

Merleau-Ponty observes that the kind of beings that we prejudicially discount as ‘not like us’ fail to register as fellow embodied subjects. As such, their behaviour is reduced to ‘blind mechanism’ rather than endowed with meaning (Merleau-Ponty [1948] 2008, 44). Their communicative practices are downgraded to that of unintelligent animals, whose gestures are instinctual, behavioural responses rather than communicative. In this sense, the person with language-inhibiting dementia is ‘dishonoured’ in the deepest possible sense (Fricker 2007, 46).

I suggest that we identify a further primary harm that arises from non-verbal testimonial injustice that concerns an obstruction in the pursuit of truth. As the communicative gestures of non-verbal people are not taken seriously, they are excluded from fundamental exercises of informational exchange, informational exchanges that play an essential role in creating a collective social understanding. As non-verbal, they are treated as though they have nothing to contribute to the shared pool of knowledge. Such exclusion from meaning-making is known as hermeneutical marginalisation (Fricker 2007, 152). Due to such hermeneutical marginalisation, we lack a proper understanding of those with advanced dementia (Petherbridge 2019; Zeiler 2014). This is detrimental not only to those who suffer from language-inhibiting dementia, as they are wrongly understood to be lacking personhood, but it also robs society in general of a proper understanding of an ever-growing group of people. Thus, non-verbal testimonial injustice elicits a broader, epistemic harm by thwarting knowledge production.

In turn, the person with language-inhibiting dementia is vulnerable to secondary harms. For one, non-verbal testimonial injustice may impact pain management. Studies show that dementia care staff frequently overlook non-verbal communications of pain (NAPP 2014). CQC recorded such instances where ‘Medical notes recorded a need for pain assessment … their daily summary recorded episodes of agitation and shouting out, but there did not appear to be any consideration that this may have been related to pain’ (2014, 20). In a further example ‘one person was holding their head and scrunching their eyes closed [indicating they had a headache]. Although a member of staff had just completed the medication round in the lounge area, this person’s pain had not been identified’ (CQC 2014, 20). A further example of physical suffering caused by non-verbal testimonial injustice is observed by Cameron et al. (2020, 1373): ‘The resident had been gesturing towards her lower body over a period of several days. It had nonetheless taken staff even longer to establish she had been in physical pain’. As such, non-verbal testimonial injustice leaves people with dementia vulnerable to prolonged physical suffering.

A further secondary harm of non-verbal testimonial injustice may be self-silencing. If a person is treated as though their communicative practices are meaningless, they may stop attempting to communicate altogether. As Fricker observes, persistent testimonial injustice may cause the marginalised subject to downgrade their own epistemic status to match the credibility deficit attached to their identity in a kind of self-fulfilling prophecy. Consequently, through the internalisation of systematic testimonial injustice and the looping effect of downgrading one’s epistemic status, the marginalised subject may eventually silence their own communicative practice. Once the subject has developed inhibited epistemic confidence, ‘the underconfident subject will tend to back down in the face of challenge, or even at the very prospect of it […]’ (Fricker 2007, 50). As such, the marginalised subject would eventually exclude themselves from a communicative exchange, having harboured the belief that they lack the epistemic ability to contribute. According to a study conducted by Fauth et al. (2020), when staff failed to engage with residents the residents were 64% more likely to remain in a ‘neutral’ state. This neutral state is described as a non-communicative ‘wakeful disengagement [which] may be concealing negative effects such as sadness’ (760). Self-silencing such as this may have especially disastrous consequences for those with dementia as studies show that without interpersonal interaction ‘a person is likely to decline and retreat, possibly into vegetation’ (Kitwood 1997, 20).

## Non-Verbal Testimonial Justice

Thus far, I hope to have convinced the reader that we ought to expand the scope of ‘testimonial injustice’ to encompass all communicative practices, including those that are non-verbal. If we fail to do so, a large portion of non-speaking people will continue to be excluded from epistemic practices. In the literature on dementia, there are various suggestions for the implementation of ‘dementia-friendly healthcare’, the purpose of which is to ‘[promote] the inclusion of people living with dementia and their carer in treatments, care decisions and discussions, with the aim of improving outcomes for the patient and carer’ (Handley, Bunn, and Goodman 2017, 2). The corrective measures proposed include not only training carers to treat dementia patients with greater empathy, but also improving working conditions for carers; alleviating time constraints for instance would increase the attention carers could give to patients. In what follows, I will focus on the corrective measures proposed by Fricker to overcome testimonial injustice and adapt them to be fit to tackle cases of non-verbal testimonial injustice too.^10^

To repair malfunctioned testimonial sensibility, Fricker (2010, 164) proposes that this perceptual faculty ought to be ‘trained’ through an awareness of the ‘dissonance between our standing beliefs in relation to speaker trustworthiness on the one hand, and our spontaneous perceptions of speaker trustworthiness on the other’.^11^ Here, Fricker calls for a reflection on our implicit prejudicial beliefs and the ways in which they conflict with our perceptual judgments of marginalised groups. Imagine a person who has been raised to hold the spurious belief that a certain race is epistemically suspect. Consequently, testimony from people who belong to this race does not appear ‘epistemically loaded’ when perceived through a biased testimonial sensibility. Over time, this belief may stand in opposition to the perceptual judgement that such ideas are unfounded and racist. Through this self-reflection, the person will wrestle with these two conflicting notions, and Fricker is optimistic that the perceptual judgement will triumph over the prejudicial belief:

Once light has dawned for a hearer, she will come to find that sometimes her experiences of testimonial exchange are in tension with the deliverances of the sensibility she has passively taken on, in which case responsibility requires that her sensibility adjust itself to accommodate the new experience. (Fricker 2007, 83)

For Fricker, by consciously reflecting upon and rejecting prejudicial beliefs that contradict our lived experience, we may be able to develop a more epistemically just outlook. The testimonial sensibility undergoes a kind of ‘gestalt switch’, whereby those excluded from this perceptual faculty now register as epistemically charged (2007, 84).

The same kind of ‘gestalt switch’ is needed to allow the non-verbal gestures of people with language inhibiting dementia to register as ‘meaningful’ within, what I call, one’s ‘communicative sensibility’. When interacting with a person who cannot communicate verbally, one ought to ‘shift intellectual gear out of spontaneous, unreflective mode and into active critical reflection’ to discard the prejudicial attitudes that skew how the gestures of people with dementia are interpreted (Fricker 2007, 91). An example of such trained communicative sensibility can be found in the example of Gladys and Naomi: Naomi is sensitive to Gladys’s non-verbal communication because she exercises ‘a basic openness to others’ (Zeiler 2014, 136). Petherbridge builds on this idea through her account of ‘embodied vulnerability’, a kind of ‘interpersonal recognition’ one facet of which is ‘a psychological openness that affirms the individual through her relations to others’ (Petherbridge 2019, 320). The gestures of a person with language inhibiting dementia may differ from people who can communicate verbally- such as Gladys banging on the side of her chair. However, through a more responsive ‘communicative sensibility’, such gestures register as meaningful.

First, Fricker (2007, 96) proposes that one’s credibility judgement becomes less susceptible to prejudicial influence through habitual interaction with the marginalised knower in question: ‘an initially socially loaded accent gets normalized with habituation; a socially alien conversational style becomes familiar; the colour of someone’s skin becomes irrelevant; their sex no longer impinges; their age is forgotten’. So too, one’s prejudicial attitudes toward people with dementia that portray them as ‘child-like’, ‘stupid’ or ‘not all there’ may conflict with one’s experience of people with dementia as coherent, thinking, feeling beings just like oneself. Through this ‘gestalt switch’, one is likely to set aside one’s prejudicial attitudes favouring empirical evidence to the contrary. Therefore, while the gestures of a person with language inhibiting dementia may initially appear senseless, in time and through familiarity, the prejudicial cloud evaporates so that they can clearly perceive non-verbal expressions as meaningful.

What about epistemic agents who do not have regular contact with a non-verbal person? As previously discussed, such marginalised knowers are subjected to non-verbal testimonial injustice not only by people they are in frequent contact with but also (and perhaps more commonly) by people they have infrequent or one-off interactions with. This could include family members who rarely socialise with them, a GP they infrequently see, a one-off interaction with a police officer, etc. While these interactions may be rare for the dominantly situated epistemic agent, for the marginalised person in question these systematic, independent cases of non-verbal testimonial injustice make up the overall wave of credibility deficit that slowly erodes their epistemic status. To avoid such one-off interactions of credibility deficit toward all marginalised groups, Fricker (2007, 96) proposes we ought to aim for ‘full possession of’ the virtue of testimonial justice: ‘ideally hearers will possess the virtue entirely spontaneously and immediately- without any prejudiced first impression having to melt away, and without any active reflection, imitation, or rehearsal’. By cultivating this virtue, correcting prejudicial attitudes in advance of a marginalised speaker’s testimony becomes second nature. In light of my argument for non-verbal testimonial injustice, I propose a virtue of *communicative* justice is more likely to capture all forms of testimonial injustice. To achieve communicative justice, one must train their communicative sensibility to be immune to the impact of identity prejudice on all communicative practices- verbal or otherwise. By cultivating the virtue of communicative justice, one’s communicative sensibility will be receptive to all kinds of expression from the most marginalised in our society. By broadening this corrective measure to include non-verbal expression, we are more likely to achieve ‘ready-corrected credibility judgements’ that encompass all forms of communicative practice (97).

Once non-verbal testimonial justice has been cultivated and a person with language inhibiting dementia is correctly perceived in epistemic colour, a two-way interpersonal interaction can occur. Most people with language inhibiting dementia not only struggle to *communicate* verbally but also struggle to *understand* verbal communication. For this reason, it is vitally important that we pay close attention to our gestural vocabulary when communicating with people who have language inhibiting dementia. To engage with a non-verbal person, one must be particularly attuned to one’s own facial expressions, eye contact, bodily contact, posture, hand gestures and proximity to the other person (Vries 2013, 33). Through heightened attention toward gestural communication, we can attain a shared ‘*critical openness’* between the bodily expressions of myself and the other person (Fricker 2007, 66). This way, we can achieve what Petherbridge calls a ‘mutual vulnerability’ which facilitates ‘a space of mutual openness’ and ‘enables family members and carers to participate in interactions that enable alternative forms of expression and communication, such that both parties in the relation or interaction are transformed’ (Petherbridge 2019, 322). Thus, through a properly trained communicative sensibility, the non-verbal knower and the verbal knower can hone ‘critical reception to her interlocutor’s world’ (Fricker 2007, 66).

## Conclusion

I end this section with the following quote from Fricker, whereby she firmly defines the limits of testimonial injustice:

The phenomenon I call testimonial injustice is not in fact confined to testimonial exchange, even allowing that we intend testimony in its broadest sense to include all cases of telling. Prejudicial credibility deficit can, after all, occur when a speaker simply expresses a personal opinion to a hearer, or as a value judgement, or tries out a new idea or hypothesis on a given audience. But telling is the parent case of testimonial injustice, since the basic wrong of testimonial injustice is the undermining of the speech *qua* knower, and, while other sorts of utterance may communicate knowledge, it is distinctive of telling that conveying knowledge is its most basic and immediate point. (2007, 60)

I argue that this conception of testimonial injustice is too restrictive. Limiting knowledge exchange to ‘telling’ is to exclude all the vital communicative practices used by people who can only express knowledge non-verbally. So too, if we consider the broad range of ways in which we come to register others as ‘knowing’ subjects, it is apparent that evaluating acts of ‘telling’ through our ‘*testimonial* sensibility’ is far too narrow a concept. This ‘certain sort of social perception’ registers people as knowers not only through *hearing* testimony but also by *seeing* embodied behaviour. This is because meaningful communication can occur through non-verbal expression.

This paper has developed an account of non-verbal testimonial injustice that I hope will expand the discourse on epistemic injustice beyond the wrong suffered by *speaking* subjects, to include those who cannot communicate verbally. Drawing on Merleau-Ponty, I understand bodily gesture to be a form of meaningful expression that ought to be considered just as valuable as speech expression and just as vulnerable to epistemic injustice. By broadening Fricker’s concept of ‘testimonial sensibility’ to ‘communicative sensibility’, we can see how non-verbal marginalised knowers can fail to show up as epistemically charged. To correct such non-verbal testimonial injustice, I propose that ‘communicative sensibility’ should be trained to encompass all forms of communicative expression from all marginalised knowers. People with language inhibiting dementia provide a paradigmatic example of non-verbal testimonial injustice, and I hope to have brought to light the previously obscured injustices they suffer.

This essay serves as an initial step in expanding the concept of testimonial injustice so that we can take seriously a wider range of communicative practices. While initially counterintuitive, I have demonstrated that even those who cannot speak can be epistemically silenced in a crucial sense. In contributing this term to the literature, there is scope for a new set of conversations in the field of social epistemology concerning the injustices suffered by those who are quite literally voiceless. I hope this paper will offer inspiration for future research into other forms of non-verbal epistemic injustice such as hermeneutical injustice, epistemic silencing and wilful hermeneutical injustice (Pohlhaus 2012). Beyond academia, the concept of non-verbal testimonial injustice may clear a path for greater epistemic sensitivity toward those who communicate in non-conventional ways, including some of those most epistemically marginalised in our society.

## Notes

1. While Glazer claims that ‘Facial expressions and tones of voice are the best examples to keep in mind’, he fails to provide an example where one’s facial expression may be met with ‘emotional misperception’. One example may be that of what is culturally referred to as ‘resting b*tch face’ (RBF): RBF is ascribed to women with a neutral facial expression, incorrectly interpreted as being stern or unhappy. This ‘emotional misperception’ is born from a sexist expectation for women to emote in more positive ways than their male counterparts, most notably to smile (Ahmed 2010).

2. Glazer does briefly mention that this ‘emotional misperception’ can take the form of a ‘false negative’ where ‘B expresses an emotion, yet A perceives B’s behavior as emotionally neutral’ (2019, 60). While Glazer mentions emotional expression as being wrongly interpreted as ‘emotionally neutral’ it is unclear what ‘emotionally neutral means’ (most likely, behaviour devoid of emotion) and the concept is not explored in detail. This paper is specifically dedicated to exploring cases in which a non-verbal expression is taken as meaningless. This paper is not claiming that non-verbal expressions are taken merely as neutral, but non-meaningful in a broader sense.

3. This is by no means the extent of Merleau-Ponty’s argument for intersubjectivity, the complex arguments for which will not be explored here. For a detailed account of Merleau-Ponty’s defence of intersubjectivity in the face of the problem of other minds, see Romdenh-Romluc (2010).

4. It is worth observing that the examples I have used thus far include meaningful gestures that communicate information both intentionally (such as when the man nods and when you point) and unintentionally (such as through the man’s facial expressions). Although intentional gestures would be more analogous to the kinds of intentional speech acts with which Fricker is concerned, both forms of gesture (unintentional and intentional) register as meaningful through the lens of a well-trained ‘communicative sensibility’. Whether the gesture is intentional or not, the person is registered as ‘epistemically loaded’. For this reason, I will not distinguish between intentional and unintentional gestures here.

5. I acknowledge that there may be something immoral about not recognising an animal, especially an intelligent animal, as a ‘knower’ or as ‘epistemically charged’. However, for the purpose of this argument, I will reserve non-verbal testimonial injustice to an injustice inflicted upon humans.

6. It is important to distinguish between linguistic and non-linguistic non-verbal people here, as I am hesitant to include people who use sign language under the umbrella of non-verbal testimonial injustice. People who rely on sign language (such as people from the Deaf community) are, of course, vulnerable to epistemic injustice. This has been well demonstrated by Scully, who draws out the testimonial and hermeneutical injustice systematically launched at the Deaf community (2018). Scully does not distinguish this epistemic injustice as importantly distinct just because it is sign language rather than spoken language. This is supported by the literature on sign language, which clearly distinguishes sign language from the kinds of gestures I discuss in this paper (Kendon 2015). Sign language is a recognised set of languages with its own grammar and lexicon depending on the region. If a person were to dismiss the sign language of someone from the Deaf community, this indeed would be a case of testimonial injustice (Scully 2018). However, as is suggested in Scully’s work, the testimonial injustice subjected to people who use sign language seems closer akin to speaking subjects who are excluded from epistemic agency. For this reason, I do not discuss people who rely on sign language in this paper.

7. An anonymous reviewer has observed that (neuro-normative) pre-linguistic infants could be vulnerable to non-verbal testimonial injustice. Although pre-linguistic infants can of course suffer injustices, I have reservations that pre-linguistic infants can suffer from *epistemic* injustices as it is unclear whether they qualify as epistemic agents. While I am open to the idea that pre-linguistic infants could suffer epistemic injustice, further research is required that cannot be conducted here. Due to the infantilisation of people with autism, intellectual difficulties and dementia, I also think it is important not to equate their epistemic capacities to that of pre-linguistic infants. Thus, pre-linguistic infants shall be set aside. It is worth noting that children can be subjected to epistemic injustice, as explored by Carel and Gyorffy (2014) and Burroughs and Tollefsen (2016). However, thus far pre-verbal infants have not been explored. The concept of pre-linguistic infants is also distinct from non-verbal children with autism.

8. It is important to note that other intersecting factors (such as gender, race and economic fragility) would also play a role in the identity prejudice attached to people with dementia.

9. It is unclear what group Merleau-Ponty is referring to here; whoever it may be, this term is prejudicial.

10. In future work, it would be fruitful to look beyond Fricker’s account of testimonial injustice to consider other ways in which this unique form of injustice could be overcome.

11. Fricker refers to this dissonance as a ‘cognitive dissonance’ (Fricker 2007, 91). However, an anonymous reviewer has pointed out that this is a superficial understanding of ‘cognitive dissonance’ compared to that found in the psychological field. To avoid misrepresenting ‘cognitive dissonance’ further I avoid using this term, and instead describe the act of self-reflection upon prejudicial beliefs that is central to Fricker’s argument.
